# Narratives in the medicolegal field from the perspective of physicians involved in medical dispute mediation meetings in Taiwan

**DOI:** 10.1016/j.heliyon.2023.e13716

**Published:** 2023-02-13

**Authors:** Po-Yi Chen, Chung-Pei Fu, Chih-Chia Wang

**Affiliations:** a Center for Medical Humanities Education, School of Medicine, National Defense Medical Center, Taiwan; b Department of Occupational Therapy, College of Medicine, Fu-Jen Catholic University, Taiwan; c Department of Family and Community Medicine, Tri-Service General Hospital, National Defense Medical Center, Taiwan

**Keywords:** Narrative, Evidence, Mediation meeting, Polyphony, Medical treatment

## Abstract

Medical treatment and narratives are interrelated. We examined this interrelation by evaluating the medical dispute mediation system in Taiwan. We conducted 16 semi-structured interviews with legal and administrative specialists in medical mediation and physicians involved in mediation meetings. The interview data were reproduced into almost verbatim text for coding and analysis. We examined how narratives were discussed in the field of medicine and identified two approaches to narratives. One was the narrative from a patient's storytelling, that is, narrative-based medicine. The other was the narrative of medical staff, which included shared decision-making and decision aids. Discussions of these approaches revolved around the avoidance of conflicts during medical treatment. However, knowing how to handle unsuccessful medical treatment is crucial. By applying polyphony in narratives, physicians can comprehend the role of narratives in unsuccessful medical treatment, helping themselves to practice how to develop narratives to communicate with patients and their surrogates when encountering any difficulty in different stages of medical treatment.

## 1 Introduction

Narratives and unsuccessful medical treatment are considered interrelated concepts in the medicolegal context. However, discussions on these concepts have largely focused on the applications of interdisciplinary training through digital representations in the classroom [1] or learning outside the classroom [2]. Moreover, studies have focused on learning outside the classroom in the field of medicolegality in law schools [3,4]. Although the amount of medicolegal research on interdisciplinary teaching is increasing, that on unsuccessful medical treatment and narratives remains scant. Therefore, this study evaluated the interrelation between narratives and unsuccessful medical treatment from the perspective of the medical dispute mediation system in Taiwan by conducting interviews with medical (major), legal, and administrative specialists. These interviews covered disputes among physicians, legal workers, and patients, thus providing a favorable perspective on medicolegal discussions.

The approach used in this study to examine the interrelation between narratives and unsuccessful medical treatment in the medicolegal context differs from those used in other related studies. Medicolegal discussions primarily focus on medical malpractice and its ensuing legal problems or the institutionalization of medical mediation [[5], [6], [7]]. Studies conducted in Mexico and Turkiye have investigated the interrelation between medical malpractice and the law and demonstrated the formation of medical dispute systems in different countries [8,9].

A study conducted in Taiwan used quantitative approaches to evaluate unsuccessful medical treatment in the medicolegal context [10]. These approaches are being increasingly used, especially after the passing of the Medical Malpractice Law in the third reading by the Legislative Yuan and its promulgation in 2022. However, after the institutionalization of medical malpractice, qualitative approaches should be employed because they can improve the complexity of humane interactions between physicians and patients, providing multiple interpretations of unsuccessful medical treatment. In Taiwan, a medical mediation meeting begins with in-hospital negotiation among physicians, patients, and a trained third party. If no agreement is reached during in-hospital negotiation, patients opt for medical dispute mediation meetings that are conducted either in the healthcare department or a court with the assistance of medical and legal mediators. The medical agreement is achieved to avoid litigation. Appendix A presents an overview of the medical mediation system and meeting process before the promulgation of the law.

While conducting semi-structured interviews in this study, we realized the multiple essences of unsuccessful medical treatment and thus applied the literary concept of polyphony for analysis.

## 2 Method: semi-structured interviews based on a discussion of constructivist grounded theory

Grounded theory is a qualitative methodology that was first used in the 1960s and then developed into constructivist grounded theory in the 1990s. Since then, constructivist grounded theory has been used more frequently to evaluate the participation of a researcher or a research group in their research. A researcher conducting research and composing narration becomes the chief narrator and is regarded as the contributor to content analyses [11,12]. Moreover, some scholars were inclined to this constructivist perspective but considered it differently: they emphasized the importance of the concept of abduction which sought a situational fit [13]. The vast application of grounded theory in different study fields led to interpretations that were interrelated to spatial and temporal contexts. In other words, the application of a theory enables scholars to discover a phenomenon from both academic and personal perspectives. This study was inspired by the discussion of the aforementioned studies on constructivist grounded theory. Moreover, we included a limited sample size in this study because of the sensitive research topic. Thus, we conducted semi-structured interviews to contextualize the medical dispute mediation system in Taiwan.

Semi-structured interviews (SSIs) are a specialized methodology developed in other studies [14,15]. Our methodological procedure involved the following steps: (1) preparing a draft or an outline for interviews, (2) arranging purposive sampling and conducting interviews with specialists, (3) preparing almost verbatim transcripts and establishing a codebook, and (4) conducting a qualitative content analysis [16].

### 2.1 Outlines

This study was approved by the Institutional Review Board of Tri-Service General Hospital (TSGHIRB No.: 1-108-05-062), and informed consent was obtained from all interviewees in this study. Before the interviews, we sent the interview outlines to the interviewees and informed them of the recording and interview procedures. We conveyed information on general procedures, including the need to provide consent, the anonymization process, and the right to withdraw from the study. After the interviews, we explained to them the data handling procedure, which included sending recordings to a typing company through Dropbox by invitation with encryption; no identifying information was provided to the company. After receiving transcripts from the typing company, we deleted the uploaded recordings.

In the first year of our study, we prepared an outline for the SSIs; however, the interview questions could not be framed using this outline. This outline consisted of six questions related to four themes, and our purpose was to comprehend how interviewees conceived medical mediation before and after their participation. The first question was related to personal involvement in medical mediation, the second and third questions were related to personal viewpoints on the procedure of the medical mediation system and two mediators (medical and legal mediators), the fourth and fifth questions related to the relationship between applicants (who usually were patients) and the other party (who usually were physicians), and the final question was used to determine the participants’ perspective on medical humanities. In the second year, we included eight questions that were based on the outline developed in the first-year outline to gain deeper insights into the medical perspective.

By following this procedure, we not only determined how experienced physicians approach this system but also identified variations in their viewpoints.

### 2.2 Purposive sampling and interviews with specialists

We conducted 16 interviews with specialists in the fields of medicine and law during our 2-year research and particularly emphasized medical specialists in the second year. We invited two types of specialists: (1) those who were licensed as a physician or legal specialists and had experience in the medical mediation system as (non-licensed) mediators at the same time in the past 3 years and (2) physicians who were involved in unsuccessful medical treatment and thus in a medical mediation or litigation in the past 5 years. All medical interviewees were licensed physicians and some of them had a law license (Appendix B). Before sending an invitation to arrange an interview, we asked our potential interviewees about their intentions. If they refused to participate in the interview, we did not send any invitation. Therefore, our invitation reply rate was 100%.

After arranging the interview date, we electronically sent our outline to the interviewees. We initiated the interview by explaining the pronunciation of the Institutional Review Board (IRB), discussing personal experiences with medical mediation meetings, and then randomly developing topics depending on the interviewees’ responses.

### 2.3 Verbatims, codebook, and qualitative content analysis

After obtaining the interviewees’ consent, we recorded the interviews on a laptop and reproduced almost verbatims in Chinese. These reproductions were prepared with the assistance of a typing company certified by the Taiwanese government and sent through Dropbox, and every record sent to this company was deleted from Dropbox once the almost verbatims were reproduced. After we received the almost-verbatim records, our research team listened to them to evaluate their accuracy. Each almost-verbatim text was coded two times.

Our analyses were based on these almost-verbatim texts, and they were translated by a translation company. Because of differences in the backgrounds of the interviewees, their approaches to the themes and the development of the topics in the interviews varied, providing opportunities for discussion. Furthermore, our coding was based on the interviewees’ participation experience and their interpretations of the meeting, including how they conceived the medical mediation meeting. This conception was better illustrated through the lens of polyphonic narratives, allowing us to probe into the viewpoints of the involved physicians.

### 2.4 Polyphonic narratives

Narratives and polyphony might be interrelated because polyphony relates to interactions among individuals and thus unsuccessful medical treatment and its ensuing mediation and litigation. Studies on polyphony have been conducted in the field of medicine. Renedo discussed the complexity of positioning an individual about others and society, and [17]; (p. 438) reported three approaches that can be used to understand clinical reasoning, highlighting the essence of a polyphonic manner.

Unlike Koufidis, who focused on the relationship between polyphony and multiplicity, [18]; (pp. 12.7–12.9) indicated that Bakhtin described polyphony as the complexity of the characters in one of Dostoyevsky's novels and later as the dialogical self, demonstrating that individuals interact with each other and form their worldviews. Polyphony is the process of the realization of the self and the knowing of other individuals. However, another interpretation of polyphony is more suitable for our research.

Cieply noted how silence was encoded within polyphony based on *Brothers Karamazov* (1879–80) by Dostoyevsky (1821–81). [19]; (p. 101) indicated that silence has two basic meanings in 19th-century Russian literature— the absence of speech (молчание) and a general noiselessness (тишина). Cieply focused on the absence of speech and demonstrated two types of expressing silence in the novel. The first was redactorial silence [20]; (p. 681), which referred to the excision of the frequent connotation in the author's correspondence, “*The thought, uttered, is a lie,*” to the publication of the novel. This excision created “*an extratemporal creative space in which the thought can be uttered and retracted without taking false, finalized form*.” The other was structured silence [20]; (p. 682), which referred to the deliberate silence that the author wrote in conversations between characters in the novel, urging the development of the plot. Cieply [682] concluded that by applying these two types of silence, the author accomplished storytelling without compromising the inner word that the polyphonic novel was devised to convey*.*

From our viewpoint, the accentuations of authorial silence in the polyphonic novel are helpful for understanding narratives in the medical mediation meeting. Narratives can consist of unspoken components, and this unspoken technique can help plot development.

## 3 Results

We used an interdisciplinary methodological approach to evaluate the medical dispute mediation system. First, the discussion on constructivist grounded theory emphasized the importance of the author. We examined this importance by conducting semi-structured interviews. Finally, we employed polyphonic narratives to verify our study findings. We speculate that this methodological approach can efficiently present our research findings In the result, we focus on how polyphonic perspectives are addressed and how polyphonic narratives are presented.

### 3.1 Beyond medical mediation meetings

Written materials, namely (1) medical case reviews written by a third-party physician and read in advance by a medical mediator and (2) mediation settlement agreements for the two parties, were frequently mentioned. The goal of a medical case review is to comprehend the context of unsuccessful medical treatment. A medical case review is conducted only in the health-care department in advance, and the testimony of a medical expert witness is provided in court.

A medical case review can be used in a medical mediation meeting in the form of a narrative of a medical mediator. Unlike medical records, which are documents with a patient's medical history in chronological order, a medical case review is a narrative consisting of scientific data (e.g., computed tomography [CT] or blood test reports) and involves personal storytelling by a third-party specialist. As indicated by M14 [5], a medical case review was first regarded as a professional analysis (專業意見) written anonymously by a physician. This physician should have specialization in the related field of the unsuccessful medical treatment, not be involved in the treatment, and not be the medical mediator of the same case.

M14, M01, and M10 mentioned the importance of a medical case review in terms of the perspective of the medical mediator. M10 indicated that if a medical mediator receives a case review from the health-care department, then this mediator can ask the medical party for supplementary documents with the assistance of the health-care department. The document can be a pathology or CT report that has not yet been provided to a medical mediator. Through this request, the medical party receives an opportunity to reevaluate the overall situation:

During the process of providing supplementary documents, the hospital or physician involved is asked to think about why they were asked to provide supplementary documents. For instance, I asked the physician to confirm the time at which the CT report was generated. In this case, the hospital or physician would consider why a CT report is required. (M10-P7)

M10 asserted that a medical mediator can understand an unsuccessful medical treatment by analyzing documents and determining individual accountability:

We communicated with the hospital regarding some problems with the standard operating procedure, such as when the report would be generated and when the physician would see the report. If there is no report, the physician can only deal with the situation at hand. (M10-P09)

Analyzing the medical case review is difficult, even for an experienced medical mediator. M01 indicated that asking for advice from other medical experts in the corresponding subspecialty becomes necessary:

If the mediator cannot understand the medical case review, they may directly ask the department secretary or director to not reveal the details of the case to anyone. (M01-P17)

M01 mentioned that the analysis of the medical case review can indicate the point of no return, an unspecialized term that was only revealed in M01's interview, defining it as the progression of a disease to an extent where everyone can see it but it has not yet been diagnosed. In this case, something may be wrong with the diagnosis (M01-P11). However, during a medical treatment, a patient may be transferred from a medical setting to another one, causing more difficulty in demanding accountability from specific medical staff. M01 noted that finding the point of no return can help delineate accountability:

I only look for the point of no return, which I mentioned earlier. A physician should be able to recognize the symptoms of a patient’s disease at that time and take prompt action. Therefore, I evaluate the patient’s medical records to determine that time point. (… …) if the medical record shows that you have noticed the symptoms, then everything is fine. However, if you did not notice the symptoms and you didn’t examine the patient’s condition when you should have, why not? How are you going to solve the medical dispute? If you did not examine the patient, you are at risk. You’ll have to discuss how you plan to resolve the dispute with the hospital and then talk with the patient’s family. (… …) Most records are now digital, and you can just print them out. However, some crucial handwritten information may be omitted. These pieces of information cannot be printed if the hospital did not archive them. Therefore, the physician should make up for these missing pieces of information/documents. Without such documents, the hospital is at a considerable risk. (M01-Pp.20-21)

The pursuit of the point of no return indicates that revealing the facts through written evidence is necessary. Moreover, this pursuit prevents medical staff in the medical party from being in an echo chamber. When medical treatment is unsuccessful, medical staff are more inclined to believe their colleagues, whose testimony can be plausible, as M01 mentioned in the interview:

When I showed the medical records to the physician involved, he was shocked. He thought it was fine because people were pampering and protecting him in the hospital, but that wasn’t the case. When you step out of your comfort zone and confront us (mediators), we provide you with facts that we observe as outsiders. In fact, all team members (the physician and his colleagues in the department) were shocked when they realized how outsiders viewed the case. Because I have no emotional connection to you, I am responsible for only telling the truth, which makes me an impartial third party. (M01-P05)

This delineation of accountability is not a considerable concern to the medical party, especially not to clinical physicians. In our interview with M02, who was informed to attend the mediation meeting because of the alleged malpractice, a common scenario was mentioned:

Over the course of those 2–3 weeks, I was under high stress because I did not know what mediation was, who would be involved, and what the setting would be. I think the stress came from uncertainty. I also didn't know the patient's attitude and demands in advance and only found out about his requirements at the mediation site. I could only prepare for the mediation, such as by bringing papers that I showed him at first and the literature I found and then explaining in detail why I chose to administer this medication. (M02-P3).

M02 did not understand what mediation meetings are. After receiving the notice to attend the mediation meeting, M02 attempted to persuade the patient on an academic basis. This did not have any effect, and at the end of the meeting, M02 agreed to cover the patient's medical expenses. Unable to comprehend the patient, M02 was trapped in the quagmire of the medical profession, expecting that the patient would comprehend the specialized medical knowledge and appropriately react even without knowing this profession.

This cognitive gap between the expertise and layman (i.e., a physician and a patient in our research) was a frequently-neglected perspective when physicians communicated with their patients. Furthermore, M02 did not realize the difference between a mediation meeting, where the physician and patient should reach a medical dispute agreement, and a medical setting, where the physician and patient intended to reach a medical consensus, and wrongly believed in the patient's ability to comprehend the situation instead of believing himself to be intellectually superior to the patient. Nevertheless, as M02 stated in the interview, the patient at that time did not believe in (Western) medicine, resulting in the failure of the mediation meeting:

However, after the patient was discharged from the hospital, he mentioned that he wanted to seek assistance from a traditional Chinese medicine (TCM) practitioner, and when he returned to the clinic, he told me that his TCM practitioner told him to stop taking all Western medicines. He wanted to see how bad his condition could get, and I agreed. Later, the patient had a severe outbreak, and he got his condition under control through TCM. When his condition gradually stabilized, he argued that it was my Western medicine that had caused the outbreak of his pustular psoriasis. (M02-P04)

M02 was unaware of the shift from a medical setting to a mediation setting, disrupting the effort to bridge the cognitive gap between the two parties.

Once accountability is delineated, a mediation settlement agreement can be accomplished. However, if no mediation agreement is established in the health-care department after one party (often the patient) enters litigation, a mediator participating in the mediation meeting of the health-care department can receive a summon of witnesses from the court. M05, a lawyer specializing in mediation study, mentioned that some party members regard a mediation meeting and the failure to reach an agreement as a challenge that they would note in court. However, a medical mediation meeting may be meaningless if it is not kept secret:

Some parties may secretly record the mediation and provide it to a judge if the mediation fails, and in such cases, the judge summons the mediator to testify in court. If I did not record it, but I heard it, can I lie? No. Then what should I do? How can you let this happen? If this happens, (… …) then you will not have to conduct a mediation. Seriously, who dares? Everyone has a sense of defensiveness. Thus, as a mediator, you must help eliminate the defensiveness between the two parties. As long as the defensiveness exists, the two parties will be on guard with each other, which makes it difficult for you to help resolve the dispute. (M05-Pp53-54)

M05 asserted that the space for candid conversation is a judicial-free environment.

When mentioning personal experience, M05, a legal mediator in health-care, shared the unexpected difficulties that mediators encounter, with medical mediators being no exception. M05's insistence was representative of the spirit of medical mediation:

The judge and clerk kept calling and threatening me, saying that I would be fined for not showing up. He didn’t dare say that he wanted to arrest me, so he said he would fine me. Well, then I would rather pay the fine. (M05-P73)

### 3.2 During medical mediation meetings

Appraising the situation and determining what to say and how to express it are nonwritten problems during medical mediation meetings. M10 mentioned superficial meaning (表面意義) and hidden meaning (深層意義), noting that (medical) mediators must think beyond only discussion with two parties, especially patients and their surrogates, and consider how and why the parties expressed their thoughts:

We talk on the basis of facts and our profession. Medical problems are handled by medical professionals, and legal problems are handled by legal professionals. Nevertheless, what you see is not necessarily reality. (… …) During mediation, both superficial and hidden meanings should be considered. Generally, what they claim is superficial. Superficial meaning cannot be used to explain deeper meaning, similarly to the issue-position-interest model proposed by Lee et al. For example, the story of brothers dividing oranges is unfair. For superficial reasons, we generally think that it is fair that each person gets half. For deeper reasons, it would not be fair to divide it in half when they do not want the orange for themselves to eat. (M10-P49)

M10 mentioned the concept of the issue-position-interest model to illustrate a crucial task for medical mediators: They are required to determine what two parties may think when they provide different interpretations of a single unsuccessful medical treatment. In addition, while receiving information from the two parties, medical mediators are advised, in the words of M07, to encourage the medical party to deliberate about the course of the unsuccessful medical treatment and tactically explain it to the patient party. If the medical party cannot speak for themselves, a medical mediator can do it for the party, leading to reconciliation between the two parties based on the truth:

However, as I said, patients really want to know what happened. If the mediator has excellent communication skills, they may be able to guide the physician to reveal what actually happened. Sometimes, the physician may be unwilling to speak. As a professional mediator, if they are a medical practitioner, they may be able to help explain it more euphemistically. Sometimes, euphemisms may actually lead to the conclusion the patient wants. As mentioned before, sometimes, what the patients or families want is not money. Why would they ask for money when their loved ones are dead? Nonetheless, they may not intend to put the physician in jail. To me, medical malpractice does not make the physician a murderer. The plaintiff knows that it is absolutely impossible that a physician would kill intentionally, so he wouldn’t want the physician to be imprisoned. So, what exactly does he want? He just cannot accept the fact that his loved ones have died inexplicably. (M07-P25)

M15, the physician who was involved in the mediation meeting as the patient party, mentioned personal feelings when hearing partial comments from the legal mediator (M15 called the legal mediator “the lawyer” in the interview because the particular legal mediator was a lawyer):

I think lawyers should talk less (… …) What they talk about really makes me … (… …) I am not here to seek legal advice. Nor am I here because I think that sentence is slightly different from a general sentence. I am not trying to figure out who is going to be convicted and on what charge. Nonetheless, he [the lawyer] said, “Don’t you have the relevant knowledge? Or no legal common sense?” Listening to him only made me sadder. (M15-P15)

As a relative surrogate of the patient, M15 noted that legal mediators lecturing patient parties on the law is inadequate. Similar to the case of M02 in the meeting, the legal mediator in M15's mediation meeting was trapped in the quagmire of the legal profession. However, M15 was deeply offended, leading to the interruption of the course of the mediation meeting. A mediator must, in the words of M10, comprehend the intentions of the two parties and thus fill the cognitive gap between the two parties instead of widening it through assumptions:

You are a mediator, and we are dealing with medical cases. Legally, we are instruments for mediation. (… …) You need to let them know that you are not entirely on the other party’s side and that you are a friend to consult. This helps the mediation succeed. Of course, most importantly, during the conversation, you need to understand the actual needs of the patient and their family. The main thing with mediation is that we need to create a channel for them to slowly ease their anger and dissatisfaction because for most of them, their primary goal is not monetary compensation. (M10-P14)

Patients can make assumptions that lead to the widening of the cognitive gap. The stereotype that physicians protect one another (醫醫相護) is commonly accepted by the patient party in medical mediation meetings. In the words of M12, medical mediators must be aware of this stereotype. M12 described a personal experience as an example and provided a solution:

When it comes to medical disputes, patients often have the notion that mediators with a medical background tend to protect and shield the physicians involved. Therefore, in terms of the seating arrangement, I prefer to be seated close to the patient’s side. We tell the patients and families that we sit next to them because we are here to provide them with advice in this regard, and we can help them uphold justice. Doing so can provide them with a greater sense of security. (M12-Pp15-16)

To bridge this cognitive gap, the interviewees noted the problem of engagement. M01 noted that it is especially difficult to engage patient parties in meetings because some might have been severely affected by the loss of their family members (patients) or other factors:

(… …) The two parties come in with emotions. When they meet, they are more melodramatic and emotional in your presence and don’t tell the truth. Patients are wary of hospital representatives, so I engage with the parties methodically. Teaching engagement is crucial. At first, we need to engage the patient and their family and then follow up with hospital representatives (including the physician involved). Health professionals follow court rules, so their engagement patterns are fixed, and they are familiar with them. No matter which hospital they belong to, their engagement patterns are probably the same. However, patients and families vary. Sometimes the situation seems simple, but they do not want to cooperate, especially when they have preconceived notions. (M01-Pp15-16)

The failure of engagement could be due to, from the perspective of M15, emotional imbalances caused by patients’ relatives; M15 was unwilling to hear any defense from the other party:

I think you get even more annoyed when someone who made you angry continues to defend himself. (M15-P10)

During mediation meetings, emotional conflicts always occur. As indicated by M07, creating separate spaces for each party and encouraging them to understand each other are key to bridging the cognitive gap, resolving emotional conflicts, and reaching an agreement:

To understand how to eliminate and alleviate psychological distress, we sometimes tell patients to put themselves in the physician's shoes during the mediation process. Sometimes you have to think from a physician's perspective. For instance, even if the physician's treatment is less than perfect, they would not directly tell you “Yes, I committed medical malpractice during surgery.” If the physician is willing to compensate the patient with a certain amount of money to resolve the dispute, the patient should probably understand that even if the physician thinks they are not at fault, they still regret the outcome. Therefore, we sometimes tell patients not to force physicians into desperate situations. (M07-P36)Our results indicated that physicians' thoughts varied depending on their roles in medical mediation meetings. The deliberate reading of the medical case review and other written materials concerned medical mediators. They also accentuated meanings in narrations, attempting to achieve a cognitive balance between the involved physicians and patients. However, the involved physicians. Who may be the patient's surrogates or physicians who allegedly misconducted in their profession, were more inclined to react in the scope of their professional training by defending themselves (M02) or putting themselves in others' shoes (M15).

## 4 Discussion

An unsuccessful medical treatment can lead to a complex situation in which every individual involved offers contradictory views. The medical mediation meeting provides an opportunity for resolving these contradictions. Therefore, studying the medical mediation meeting and its participants is crucial. In this section, we reconsider the physician-patient relationship from the perspective of the medical dispute mediation system through the lens of constructivist grounded theory and polyphonic narratives. We hope to provide an insightful perspective of physicians, and this perspective can be applicable to not only physicians but also medical staff. The discussion on constructivist grounded theory highlighted the importance of an author, and polyphonic narratives concentrate on how an author composes their narrations in mind or through non-verbal gestures (directly or indirectly). These perspectives are not always in accordance with the current situation that physicians in Taiwan have encountered. In the case of M02, the patient was more inclined to TCM and M02 was unable to avoid the academic quagmire. However, standing on the opposite side of medical staff in the mediation meeting, M15 attempted to minimize the cognitive gap between their family members and the medical side and achieve a consensus proved in vain. This was mainly because of the unqualified legal mediator and the lack of mutual understanding. Although trained and experienced mediators can effectively bridge this cognitive gap, we aim to provide insightful suggestions following the current study of the physician-patient relationship.

### 4.1 Current study: narratives in medicine

Narratives often involve bilateral relationships (i.e., between a physician and a patient) in the field of medicine and are approached through various methods. For instance, some researchers have used the term “narratives” in their studies, whereas others have defined the term differently. In the field of medical education, narratives that focus on discussions between narrative-based medicine and evidence-based medicine (EBM) might change the role of narratives from patients’ perspectives.

Considering that medicine is an expanding field of study, Naylor reported that medicine is in a state of uncertainty, highlighting halfway technologies that blur the boundaries of EBM. This has also enlarged gray areas in medical treatment. [21]; (p. 841) indicated that the limitations of medical evidence continue to limit the ambit of EBM.

The definition of evidence has been reconsidered. [22]; (p. 50) reported that Kathryn Hunter questioned the definition of evidence in medical education, asserting that in addition to science, medicine can be considered a source of moral knowledge, narratives, interpretation, and practical reasoning. The role of narratives in the scope of evidence has also been discussed. [23]; (p. 395) indicated that narratives can provide different approaches to understanding evidence, enabling a patient to express their experiences to a physician in a medical setting. This helps physicians understand the individuality of a patient, provides a teaching example that can be irrelevant to an exemplary case in textbooks, and can inspire medical students and staff to approach traditional treatment through new methods. Narratives can link a patient's syndrome to a specific disease, illustrating the individual aspect and therefore the complexity of medicine and providing a new approach to medical education.

Rhodes and Lancaster [24]; (p. 238, p. 1) described another approach to comprehending evidence. The authors conceptualized evidence-making intervention and focused on the contingency of medical treatment, asserting that physicians should decide on interventions on the basis of emerging scientific signals instead of on the basis of existing ones. This not only redefines evidence but also emphasizes the importance of the contextualization of individual medical treatment. Thus, physicians should know when and how to provide the appropriate treatment for each patient with the same disease.

Evidence is related not only to the narratives of patients but also to the narratives of physicians, as indicated by Rhodes and Lancaster. Because physicians might experience difficulty in comprehending patients’ thoughts, analyzing narratives is crucial. Following the development of the concept of decision aids can be another approach for comprehending narratives.

The promotion of decision aids by Ref. [25]; (pp. 736–737) is related to the complexity of a medical treatment. The authors indicated that patients' perspectives should be considered to form an alliance with the patient and resolve the medical problem. However, [26]; (pp. 1–2) reported that although decision aids would enhance patients' understanding of personal disease and latent risk, they would limit physician-patient communication. Moreover, choice framing and evidence-based selective information may compromise the authenticity of decision aids. Thus, although patients believe that they are making choices on their own, their options are limited by the present (limited) medical evidence and based on scientific information provided by a physician. Therefore, [27]; (p. 1296) indicated that providing optimal patient care through shared decision-making involving EBM and patient-centered communication is necessary to achieve a balance between the subjectivity of a physician and the objectivity of a patient's situation.

These topics are crucial to narratives in medicine. Irrespective of the use of decision aids or shared decision-making, these topics relate to how a physician should narrate. [28]; (pp. 571–572) reported the application of prospect theory to physician-patient communication. They indicated the importance of acknowledging uncertainty and thus highlighting the applications of loss-framed messaging. Through this messaging, patients can determine latent risks. They suggested that physicians should use various strategies in their conversations with patients, including the use of personal narratives based on scientific reasoning. However, keywords such as “narratives” were not used in the article.

Narratives in medicine can be those of the patient and those of the physician. This categorization indicates that narratives are not unidirectional. In addition, they are related to the success of medical treatment. However, reading narratives in polyphonic contexts is helpful, relating to how a physician should think through an unsuccessful medical treatment and the ensuing legal treatment.

### 4.2 Narratives in medical mediation meetings

Narratives are not fixed, which became apparent through our study, and every narrator can redefine narratives as independent agents. Interactions between agents can form a broader, complicated narrative. In a medical mediation meeting, a narrative consists of scientific and descriptive components. The scientific component involves the medical reports or records of a patient. The descriptive component refers to patients’ narratives of their disease or narratives that the two parties constructed before the medical mediation meeting. Medical mediators must form a broader and more complicated narrative by reading the medical case review and comparing it with the narratives of other narrators (e.g., the two parties or their surrogates).

While forming a broader narrative, a medical mediator must have a blueprint of plot development in mind and determine what to say to which party and when to say it. Creating a hook for the plot can be difficult and identifying the point of no return in a medical case review or authorial silence during the meeting can be helpful. This can involve “finding a point of no return prior to a mediation meeting (M01-Pp.20-21) and then deciding to whom to talk and to what extent (M07-Pp.25, 36)” or “asking other physicians for advice without revealing (M01-P.17).” Although revealing the truth, which often causes controversy, is not the priority for medical mediators, alleviating the tension in relationships between a physician and a patient and achieving an inner reconciliation between the two parties are vital.

### 4.3 Insightful suggestions to physicians

On the basis of the findings of the current study on the physician-patient relationship, determining the importance of narratives is not difficult. Although we cannot judge the importance of the physician-patient relationship solely from this study, through the interviews of physicians involved in the mediation meeting (M02 and M15), practical lessons for physicians were obtained. In addition, we obtained insightful findings by conducting interviews with medical mediators.

We prefer interpreting the recent discussion on narratives in the medical field because a series of patient-centered theories can jeopardize medical professionalism. By nudging a patient or providing them with a prospect statement, physicians can instill their professional insight on the basis of both academic and empirical aids in their patients. This approach is useful and applicable to our interviewees who were involved in mediation meetings.

The perspective of medical mediators and the relationship of this perspective with polyphonic narratives should be considered to enrich the scope of this concept. Medical mediators focus on achieving a consensus between the other two sides in mediation meetings with the assistance of the deliberate readings of written materials and the comprehension of differences between superficial words and potential but unspoken meanings. This technique requires detaching themselves from the medical profession and intellectual independence to compose narrations in mind and narrate them to patients. This intellectual switch from unspoken words to spoken, from scientific profession to humane interactions, plays a crucial role in medical treatment regardless of its success.

## 5 Conclusion

We evaluated unsuccessful medical treatment by determining the perspectives of patients and physicians in medical mediation meetings in Taiwan. Interactions between a physician and a patient vary by location. Our research demonstrates that the interaction of a physician with a patient differs between a medical setting and a mediation meeting. In a mediation meeting, physicians should distance themselves from the professional sphere of medicine.

A polyphonic perspective should be adopted to explore the multilateral aspects of unsuccessful medical treatment. When interacting with patients, physicians should apply corresponding narratives to different stages of medical treatment, whether through silence or interaction, regardless of their success.

We also presented our research in a broader academic context, providing physicians with an insightful suggestion that the intellectual switch is crucial at any stage of medical treatment.

## 6 Limitations

We did not include an adequate sample size required for SSIs because of difficulties related to recruitment. The interviewees’ discussion on their experiences resulted in most physicians in the medical party and patients and their surrogates (usually relatives) feeling considerable sorrow. One of our interviewees, a physician experiencing litigation, repeatedly declared their innocence and described mistreatment from the media. In addition, because our study focused on selective but variant references in different fields of study, our research is unconventional.

## Author contribution statement

Po-Yi Chen: Performed the experiments; Analyzed and interpreted the data; Wrote the paper.

Chung-Pei Fu: Analyzed and interpreted the data; Contributed reagents, materials, analysis tools or data.

Chih-Chia Wang: Conceived and designed the experiments; Performed the experiments; Analyzed and interpreted the data.

## Data availability statement

The data that has been used is confidential.

## Declaration of interest's statement

The authors declare no competing interests.

## Figures and Tables

**Table A1:** Interviewees' backgrounds.

Interviewee no.	M01	M02	M03	M04	M05	M06	M07	M08
Professional Certification (s)	Medical	Medical	n/a	n/a	Law	Medical	Medical and Law	Law
Position	Physician	Physician	n/a	n/a	Lawyer	Physician	Lawyer	Judge
Primary role in the meeting	medical mediator	medical party	medical admin.	medical admin.	legal mediator	medical mediator	medical mediator	Legal mediator
sex	M	M	M	F	M	M	M	M
Interviewee no.	M09	M10	M11	M12	M13	M14	M15	M16
Professional Certification (s)	Medical	Medical and Law	Medical	Medical	Medical	Medical	Medical	Law
Position	Physician	Physician	Physician	Physician	Physician	Physician	Physician	Lawyer
Primary role in the meeting	medical party	medical mediator	medical party	medical mediator	medical party	medical mediator	patient party	legal mediator
Sex	M	M	M	M	M	M	F	F

## Appendix A Overview of the Medical Dispute Mediation System in Taiwan prior to the Promulgation of the Medical Malpractice Law

### 1. Overview

1-1. After the promulgation of the Medical Care Act on November 24, 1986, and the amendment in 2004, according to Chapter 8 (Medical Review Committee), Article 99, the members of the Medical Review Committee were obliged to be medical mediators.

In addition, the Department of Health-care in Taipei County, upgraded to a special municipality as New Taipei City in 2010, first conducted a medical mediation meeting with two mediators (legal and medical ones) and later became a model for other health-care departments.

1-2. Taiwan Code of Civil Procedure, Article 403-7 states that disputes arising from medical treatment “shall be subject to mediation by the court before an action is initiated.” In September 2012, the first mediation meeting with two mediators by the court was conducted in Taiwan Taichung District Court.

1-3. The government of Taiwan incorporated the two mediation systems into the Medical Malpractice Law, which passed the third reading by the Legislative Yuan in Taiwan on May 30, 2022, and was promulgated on June 22, 2022.

In 1990, the first version of the Medical Care Network was implemented. In 2013, the Medical Care Network initiated a project on the application of medical care to medical dispute resolution procedures. In 2017, the Ministry of Justice and the Ministry of Health and Welfare attempted to execute a project to standardize operation procedures for a medical mediation. Before litigation, a plaintiff (usually a patient party) should conduct a medical mediation in the health-care department. Refusal leads the plaintiff to the court system, namely a medical mediation before the litigation and the ensuing actions (mediation agreement or litigation).

### 2. Conduct in mediation meetings

#### 2-1. In-hospital negotiation

An in-hospital negotiation is conducted between medical staff and the patient after an unsuccessful medical treatment. However, in addition to medical centers, most clinics and hospitals do not have qualified training for such negotiations. Therefore, the Ministry of Health and Welfare of Taiwan launched the Triple-I Health Mediator training program in 2018 to close this resource gap.

#### 2-2. Medical dispute mediation

Normally, if no agreement is reached during in-hospital negotiation, patients opt for either litigation or mediation, seeking the involvement of third-party, medical and legal mediators. In Taiwan, medical dispute mediation meetings can be conducted in the health-care department and court, and the process of mediation meetings in the two fields is similar. Although two independent approaches for mediation are currently used, they will be unified.

In general, the steps of the mediation meeting process are as follows. (1) One of the parties, usually the patient party, applies for a mediation meeting in the health-care department or initiates litigation against the hospital or medical staff, before which a compulsory mediation meeting is required. If a meeting is conducted in the health-care department, a medical case review, which is performed by a third-party physician and cannot be included as evidence in the potential ensuing litigation, can be conducted upon request. A physician who performs the medical case review cannot be the medical mediator of the same unsuccessful medical treatment. A pre-meeting between the two mediators is usually held to reach a consensus on the event. (2) The medical party, either administrative or medical staff, is invited to the meeting, which consists of two mediators (legal and medical ones) and two parties (the patient one and themselves). In a meeting, the legal mediator opens a meeting by announcing the notice of the procedure. Then, the two mediators talk to the parties together. Only upon receiving specific requests can mediators ask for separate talks with each party. The meeting lasts between 60 and 90 min, and after this, the two parties may reach an agreement. If no agreement is reached, the patient party can resort to litigation. If the two parties fail to reach an agreement in a mediation meeting in the health-care department, a compulsory mediation meeting prior to litigation is held only upon request.

If a mediation agreement is reached, a cardinal difference exists between the two fields; that achieved in the court has (Civil) Res Judicata and that made by the health-care department has not. As the enactment of the Medical Malpractice Law (Draft) progresses, this difference will be justified to have (Civil) Res Judicata. In addition, this draft provokes the debate on the role of the medical case review; some medical and legal scholars, chiefly from the members of the Taiwan Health-care Reform Foundation (THRF), insist on the viewpoint that medical case review is professional and therefore applicable in ensuing litigation. If this medical case review cannot be a piece of evidence, the justification of bothering the third-party physicians to write it remains unclear.
